# Driving Cessation and Dementia: Results of the Prospective Registry on Dementia in Austria (PRODEM)

**DOI:** 10.1371/journal.pone.0052710

**Published:** 2012-12-26

**Authors:** Stephan Seiler, Helena Schmidt, Anita Lechner, Thomas Benke, Guenter Sanin, Gerhard Ransmayr, Riccarda Lehner, Peter Dal-Bianco, Peter Santer, Patricia Linortner, Christian Eggers, Bernhard Haider, Margarete Uranues, Josef Marksteiner, Friedrich Leblhuber, Peter Kapeller, Christian Bancher, Reinhold Schmidt, PRODEM Study Group

**Affiliations:** 1 Department of Neurology, Division of Neurogeriatrics, Medical University of Graz, Graz, Austria; 2 Institute of Molecular Biology and Biochemistry, Centre for Molecular Medicine, Medical University of Graz, Graz, Austria; 3 Department of Neurology, Medical University of Innsbruck, Innsbruck, Austria; 4 Department of Neurology, General Hospital Linz, Linz, Austria; 5 Department of Neurology, Medical University of Vienna, Vienna, Austria; 6 Department of Neurology, Konventhospital der Barmherzigen Brüder Linz, Linz, Austria; 7 Department of Geriatric Psychiatry, Landesnervenklinik Sigmund Freud Graz, Graz, Austria; 8 Department of Psychiatry and Psychotherapy, Regional Hospital Hall in Tirol, Hall in Tirol, Austria; 9 Department of Neurology and Geriatric Psychiatry, Nervenklinik Wagner-Jauregg Linz, Linz, Austria; 10 Department of Neurology and Psychosomatic Medicine, Regional Hospital Villach, Villach, Austria; 11 Department of Neurology, Regional Hospital Horn, Horn, Austria; Cardiff University, United Kingdom

## Abstract

**Objective:**

To assess the influence of cognitive, functional and behavioral factors, co-morbidities as well as caregiver characteristics on driving cessation in dementia patients.

**Methods:**

The study cohort consists of those 240 dementia cases of the ongoing prospective registry on dementia in Austria (PRODEM) who were former or current car-drivers (mean age 74.2 (±8.8) years, 39.6% females, 80.8% Alzheimer’s disease). Reasons for driving cessation were assessed with the patients’ caregivers. Standardized questionnaires were used to evaluate patient- and caregiver characteristics. Cognitive functioning was determined by Mini-Mental State Examination (MMSE), the CERAD neuropsychological test battery and Clinical Dementia Rating (CDR), activities of daily living (ADL) by the Disability Assessment for Dementia, behavior by the Neuropsychiatric Inventory (NPI) and caregiver burden by the Zarit burden scale.

**Results:**

Among subjects who had ceased driving, 136 (93.8%) did so because of “Unacceptable risk” according to caregiver’s judgment. Car accidents and revocation of the driving license were responsible in 8 (5.5%) and 1(0.7%) participant, respectively. Female gender (OR 5.057; 95%CI 1.803–14.180; p = 0.002), constructional abilities (OR 0.611; 95%CI 0.445–0.839; p = 0.002) and impairment in Activities of Daily Living (OR 0.941; 95%CI 0.911–0.973; p<0.001) were the only significant and independent associates of driving cessation. In multivariate analysis none of the currently proposed screening tools for assessment of fitness to drive in elderly subjects including the MMSE and CDR were significantly associated with driving cessation.

**Conclusion:**

The risk-estimate of caregivers, but not car accidents or revocation of the driving license determines if dementia patients cease driving. Female gender and increasing impairment in constructional abilities and ADL raise the probability for driving cessation. If any of these factors also relates to undesired traffic situations needs to be determined before recommendations for their inclusion into practice parameters for the assessment of driving abilities in the elderly can be derived from our data.

## Introduction

With few exceptions [1] previous studies reported an increased risk for car accidents in patients with dementia [2]–[4]. It has been reported that 22–46% of patients with mild to moderate dementia still drive [5]–[7]. Surprisingly little is known about factors influencing driving cessation in dementia cases [5], [8]. In addition, there exist considerable differences among countries with respect to legal regulations for the evaluation of fitness to drive in elderly people. In Finland, license renewal including medical evaluation is mandatory every five years, starting at the age of 45. Most, but not all US states tightened the licensing process for older people by shortening renewal periods and by imposing medical check-ups depending upon the license holder’s condition [9], [10]. In contrast, Austria, Belgium, France and Germany by law do not impose any regular medical assessment of driving abilities in the elderly. They grant lifelong driving permission [11], [12]. The European Council directive 2006/126/EG [13] gives general recommendations for license issuing and renewal in demented people. These require an authorized medical opinion.

Practice parameters for medical assessment are needed, but they are scarce and at this point rather rely on expert opinion than evidence-based parameter selection [14].

The Canadian consensus conference on dementia suggests cognition, function and medical status being important in the evaluation of driving abilities [15]. The American Academy of Neurology [14] bases recommendations for driving cessation on clinical dementia rating (CDR). The Canadian Medical Association [16] and the Austrian Federal Ministry for Transport, Innovation and Technology [17] consider the MMSE score as essential. In this context it is important to note that the MMSE is of dubious value because recent data assessing the predictive role of this dementia screening test on simulated car driving ability failed to demonstrate a significant association [2]. We here assessed the association between parameters which have traditionally been recommended for the assessment of driving abilities of elderly people and driving cessation in a large cohort of dementia patients who participated in a prospective national dementia registry conducted in memory clinics in Austria.

We also assessed the influence of Behavioral and Psychological Symptoms of Dementia (BPSD) as well as caregiver characteristics and strain of care on driving cessation.

## Methods

### Ethics Statement

The study was approved by the ethics committees of the Medical University of Graz, the Medical University of Innsbruck, the Medical University of Vienna, the Konventhospital Barmherzige Brüder Linz, the Province of Upper Austria, the Province of Lower Austria and the Province of Carinthia. Written informed consent was obtained from all patients and their caregivers.

### Study Population

The prospective registry on dementia in Austria (PRODEM) is an ongoing longitudinal multi-center cohort study conducted in 12 memory clinics in our country. Since 2009, 437 subjects have been included. Inclusion criteria are (1) dementia diagnosis according to DSM-IV criteria [18], (2) non-institutionalization and no need for 24-hour care, (3) availability of a caregiver who agrees to provide information on the patients’ and his/her own condition. Patients were excluded from the study if they were unable to sign an informed consent or if co-morbidities were likely to preclude termination of the study.

The study centers were situated in six of nine provinces of the State of Austria with investigators representing the specialties of neurology and/or psychiatry. Historical information and clinical as well as neuropsychological examinations were collected at baseline and every six months over a time period of two years or until institutionalization, withdrawal from the study, loss to follow-up or death. At each visit, patient- and caregiver assessments followed a pre-defined protocol, which was administered at every participating center. The baseline evaluation included patient- and caregiver demographics, duration of dementia symptoms, assessment of the patients’ living situation and resource utilization, driving status, presence of co-morbidities, recording of anti-dementia and concomitant medication, as well as extensive clinical, cognitive, behavioral and functional assessment. Caregiver burden was also assessed. Bio-banking including sampling of DNA, RNA, plasma and serum was done. MRI scans were obtained according to standardized protocols.

The current study cohort consists of those 240 study participants who ever drove a car. Alzheimer’s disease (AD), Vascular Dementia (VaD), Lewy Body Dementia (LBD), Frontotemporal Dementia (FTD) and other dementias were diagnosed in 194, 12, 11, 16 and 7 patients, respectively. Their mean age was 74.2 (±8.8) years and ranged from 41 to 100 years. There were 95 (39.6%) females. The mean duration of dementia prior to inclusion into the study was 33.0 (±24.6) months. The mean MMSE and CDR scores of patients were 22.1 (±4.5) and 0.9 (±0.6), respectively. The mean DAD score was 71.5% (±25.3%). At study entry 145 (60.4%) participants had ceased driving during the course of dementia.

In patients with possible AD, we used MRI findings to determine evidence for mixed AD and vascular pathology. From a total of 61 patients with possible AD, MRI was available in 41 subjects. Among them, 9 (22%) patients had evidence for mixed pathology.

Causes for driving cessation were obtained on the basis of information from the caregivers. They were categorized into (1) unacceptable risk, (2) involvement in a car accident, or (3) revocation of the driver’s license.

### Possible Risk Factors for Driving Cessation

#### 1.Dementia types

Dementia types were diagnosed according to the NINCDS-ADRDA Criteria for AD [19], ADDTC Criteria for VaD [20], McKeith Criteria for LBD [21] and McKhann Criteria for FTD [22]. In cases classified as having possible AD evidence for a mixed AD and vascular pathology was defined based on presence of confluent white matter changes or large or multiple cerebral infarcts including lacunar infarcts on MRI [23].

#### 2.Demographic and social factors

Age, sex, retirement- and marital status, education, occupation and living situation including assistance at home were assessed by structured questionnaires at study entry. Educational level was categorized into (1) less than high school diploma, (2) high-school diploma and (3) university degree. Occupational status was classified according to the patients’ longest occupation in life. Categories were (1) blue-collar worker, (2) white-collar worker, (3) self-employment and (4) housewife.

#### 3.Cognition

We assessed the Mini Mental State Examination (MMSE) [24] as proposed by the Canadian Medical Association and Adler et al. [16], [25], the Clinical Dementia Rating (CDR) scale [26] according to Dubinsky et al. [14] and measured cognition, function and medical status following the Canadian Consensus Conference on Dementia [15].

For assessment of cognitive functioning the “Consortium to Establish a Registry for Alzheimer’s disease (CERAD) – Plus” neuropsychological test battery has been used [27], [28]. In brief, this battery consists of ten subtests including verbal fluency, the Boston Naming Test, MMSE, a word list memory test, constructional praxis, word list recall and word list recognition. “Plus-tests” are the Trail Making Test parts A and B as well as phonematic fluency (s-words). Subtest scores were converted to standardized scores (z-scores) using the population mean and standard deviation, adjusted for age, sex, and education. Lower values indicate poorer performance with respect to the population mean.

#### 4.Function

To assess function, we applied the disability assessment for dementia (DAD) scale [29]. This instrument evaluates basic and instrumental activities of daily living (ADL), leisure activities and the patients’ ability to initiate, plan, organize and complete the specific actions. The global score gives a percentage of overall function in ADL. Higher values represent less disability.

#### 5.Medical status

Assessment of medical status included history of stroke, coronary heart disease, atrial fibrillation, venous thrombosis and major vascular risk factors including hypertension, diabetes and hypercholesterolemia. Definition of vascular risk factors followed the American College of Cardiology Foundation/American Heart Association [30], American Diabetes Association [31] and National Cholesterol education program adult treatment panel III Practice Guidelines [32]. Administered medication was recorded using a standardized questionnaire.

#### 6.Behavioral and psychological symptoms of dementia (BPSD)

BPSD were assessed by the Neuropsychiatric Inventory (NPI) [33] a scale composed of 12 sub-items including delusions, agitation/aggression, depression, anxiety, euphoria, apathy, disinhibition, lability, aberrant motor behavior, sleep, appetite and eating disorders. Frequency (1–4 points) and severity (1–3 points) of symptoms are rated. The total score ranges from 0 to 144 with higher values indicating higher behavioral and psychological disturbance.

#### 7.Caregiver related factors

The relationship between caregiver and patient was evaluated and categorized into spouse, unmarried partner, child, other relative or non-relative. The caregivers’ employment status distinguished between unemployed, fully employed, partly employed, in training, reduced employment because of care, retired or other.

Caregiver burden was assessed using the Zarit Burden Interview (ZBI) [34]. This consists of 22 questions and measures subjective burden among caregivers of patients suffering from dementia. Functional and behavioral impairments as well as the home care circumstances are addressed. A maximum of 88 points can be reached and higher values correlate with more severe burden.

### Statistics

For statistical analysis we used the Statistical Package of Social Sciences (SPSS) version 19. Between-group differences in categorical variables were compared using the χ^2^ test. Assumptions of normal distribution for continuous variables were tested with the Kolmogorov-Smirnov test. Normally distributed continuous variables were compared using the Student’s t-test and the Mann-Whitney-U test was applied in case of non- normally distributed variables. All patient- and caregiver related factors found to be associated with driving cessation at a p-value lower than 0.05 in univariate statistical analysis were then simultaneously entered into a multivariate logistic regression model to determine their independent contribution on the patients’ driving status. Variables were assessed for multicollinearity by Pearson’s and Spearman’s correlation coefficient and evaluated for exclusion if values >0.7 occurred. Odds ratios and 95% Confidence Intervals were calculated from the beta coefficients and their standard errors.

## Results

With 90.9% the highest rate of driving cessation was seen in patients with LBD. The cessation rate in AD cases was 58.2%. Similar figures were seen in VaD and FTD with 66.7% and 56.3%, respectively. The subgroup of study participants with evidence for a mixed AD and vascular pathology ceased driving at a comparable rate of 55.6%.

In 136 cases (93.8%) the cause for driving cessation was “too high risk” reported by the caregiver. Car accidents caused cessation in 8 cases (5.5%) and revocation of the driving license in only 1 participant (0.7%). As can be seen from table 1, patients who had ceased driving were older, more commonly women and retired, they more often were assisted at home and had more severe clinical dementia stages with higher disability. Their MMSE scores and CERAD-assessed constructional abilities were lower. They had more aberrant motor behavior and were more apathetic according to NPI. Additionally their caregivers’ strain was higher. No other factors including dementia type, patient- and caregiver demographics and co-morbidities were significantly associated with the driving status of study participants in univariate analysis.

**Table 1:** Significant associations between patient- and caregiver related factors and driving status: Results of univariate statistical analysis.

Variable	Driving cessation	No driving cessation	p-value
	N = 145	N = 95	
Age, yrs (median, IQR)	77(71.00–81.00)	74(67.00–79.00)	0.037†
Female gender (N, %)	65(44.80)	30(31.60)	0.040‡
Retired (N, %)	132(97.10)	80(90.90)	0.046‡
Assistance at home (N, %)	126(87.50)	73(76.80)	0.031‡
MMSE (median, IQR)	22.00(18.50–25.00)	24.00(21.00–26.00)	<0.001†
CDR (median, IQR)	1.00(0.50–1.00)	0.50(0.50–1.00)	<0.001†
CERAD Constructional practice (mean, SD)	−0.98 (1.79)	−0.13(1.51)	0.001#
NPI – Aberrant motor behavior (N, %)	34(23.40)	12(12.80)	0.041‡
NPI – Apathy (N, %)	66(45.80)	28(29.80)	0.013‡
DAD (median, IQR)	67.50(42,50–87.50)	87.00(72.50–97.50)	<0.001†
ZBI (median, IQR)	22(11.00–32.00)	13(4.00–24.00)	<0.001†

SD = Standard Deviation, IQR = interquartile range.

† Mann-Whitney-U-Test.

‡ χ^2^ Test. #Student’s t-test. Abbreviations: MMSE = Mini Mental State Examination, CDR = Clinical dementia rating, CERAD = Consortium to establish a registry for Alzheimer’s disease, NPI = Neuropsychiatric Inventory, DAD = Disability Assessment for Dementia, ZBI = Zarit Burden Interview.

Logistic regression analysis determined female gender, constructional abilities on CERAD and impairment in ADL to be the only significant and independent associates of driving cessation (table2).

**Table 2:** Associations between patient and caregiver related factors and driving status: Results of logistic regression analysis.

Variable	OR	95% CI	P-value
Age	1.048	0.986–1.114	0.129
Female gender	5.057	1.803–14.180	0.002
Retired	1.627	0.257–10.285	0.605
Assistance at home	0.721	0.234–2.225	0.569
MMSE	1.034	0.896–1.194	0.646
CDR	1.553	0.343–7.042	0.568
CERAD constructional practice	0.611	0.445–0.839	0.002
NPI – aberrant motor behavior	1.524	0.417–5.571	0.524
NPI – apathy	0.521	0.191–1.424	0.204
DAD	0.941	0.911–0.973	<0.001
ZBI	1.027	0.989–1.066	0.164

Abbreviations: OR = Odds Ratio, CI = Confidence Interval, MMSE = Mini Mental State Examination, CDR = Clinical dementia rating, CERAD = Consortium to establish a registry for Alzheimer’s disease, NPI = Neuropsychiatric Inventory, DAD = Disability Assessment for Dementia, ZBI = Zarit Burden Interview.

## Discussion

We confirm previous studies reporting that one in three dementia patients still drives [6], [35], [36]. The risk-estimate of caregivers regarding the patients’ driving abilities was the most common reason leading to driving cessation. Women and patients with poor constructional abilities and impaired activities of daily living had the highest probability to cease driving.

Others also reported that women are more likely to cease driving than men. This was observed in healthy aging [37]–[39] and dementia [5]. It has been described that women show more avoidance behavior and experience more severe stress in road traffic than men [37], [40] which might be one reason for the described gender difference with respect to driving cessation in our investigation. Factors significantly and independently predicting driving status in the current study are only partially reflected by existing practice parameter recommendations. Our findings are in line with the Canadian Consensus Conference on Dementia indicating the cognitive and functional status of demented patients being instrumental when assessing their fitness to drive. The domain most closely associated with driving cessation was visuo-constructional functioning, a finding corroborated by several studies assessing the association between visuospatial abilities, driving safety and road test performance [41]–[44]. In contrast to the recommendations of the Canadian Medical Association [16] and of the American Academy of Neurology [14] neither the MMSE score nor the CDR was independently associated with driving status in our study. Both measures were associated with driving cessation in univariate analysis, but the associations did not remain significant when test results of constructional practice, activities of daily living and behavior were included. This finding is in line with recent traffic medicine developments indicating that traditional neuropsychiatric approaches as recommended in practice parameter guidelines are not helpful, but it is rather driver behavior, insight and preserved strategic competence that are relevant [45]. Moreover, recent studies indicate that medical and cognitive screening of older drivers may even be harmful in public health terms. An Australian study [46] examined older driver fatal and serious injury crash involvement rates and determined possible associations with different licensing procedures. Older drivers in jurisdictions with age-based mandatory assessment programs were not safer than those in states without such programs. There was some indication that subjects living in areas without mandatory assessment practices had an even safer record regarding overall involvement in serious casualty crashes. These results are corroborated by another study by Siren and coworkers [47] in which the authors showed that cognitive screening of older drivers, despite intuitively making sense, failed to produce the desired benefits. After implementation of a cognitive screening program more drivers were involved in fatal accidents. This applied to subjects below and above the age of 72 years. One possible explanation was that drivers who did not pass cognitive screening shifted to more dangerous modes of transportation which made them more vulnerable in traffic.

Unlike other studies [5], [25], [35], we failed to demonstrate an independent association between age and driving cessation. However, differences in age do not seem to be the reason for that finding as the age of current sample was similar to previously published cohorts [5], [25], [35].

There was also no association between driving status and dementia type, but the relatively low number of non-Alzheimer dementias in our investigation needs to be emphasized. Our study is the first to include caregiver related factors as possible predictors of driving cessation in demented patients. The study result that the caregivers’ estimate of “unacceptable risk” was the reason to cease driving in more than 90 percent of our patients underscores the pivotal role and key responsibility of caregivers in the decision as to whether demented patients still drive. The reliability of the caregivers’ judgment regarding fitness to drive in old people is supported by a recent study by Stapleton and coworkers [48] who examined the usability of self- and proxy awareness scales for screening post-stroke patients as to their ability to return to driving. The authors showed that patient- and proxy test-scores were highly correlated with each other and with on-road test results [48]. Although in our study the decision for driving cessation depended very much on the caregiver, it was unaffected by any caregiver characteristic per se, including age, sex, relationship to the patient, employment status or strain of care. To our knowledge, there exist no numbers in the literature concerning car accidents and revocation of the driving license as reasons leading to driving cessation in demented patients. However, in our cohort, as compared to the caregivers’ risk-estimate, these factors played a marginal role, only.

A strength of our study is its prospective study design with the use of pre-defined and standardized questionnaires in a nation-wide multicenter setting.

There are also weaknesses. We included only patients who attended memory clinics and who had caregivers willing to be part of the investigation. Therefore the cohort composition may not be representative for the general dementia population. It is likely that we rather overestimated the frequency of driving cessation given the central role of caregivers in the decision-making as to whether demented patients quit driving.

We can also not exclude with certainty that the factors that influence driving cessation in dementia patients with caregivers differ from those in patients without caregivers. It is also possible that other somatic factors that were not specifically included in our analysis like motor dysfunction or visual disturbance influenced driving status. Other factors which remained un-assessed in our investigation despite they have been shown to be related to performance on structured road tests are measures of strategic and tactical thinking [49].

We consider the identification of demographic, clinical and caregiver-related associates of driving cessation in patients with dementia only the first step in the development of evidence-based practice guidelines. Future studies determining the role of each single factor or their combination as predictors of moving violations including car accidents are warranted.
